# Plexin-D1/Semaphorin 3E pathway may contribute to dysregulation of vascular tone control and defective angiogenesis in systemic sclerosis

**DOI:** 10.1186/s13075-015-0749-4

**Published:** 2015-08-21

**Authors:** Celestina Mazzotta, Eloisa Romano, Cosimo Bruni, Mirko Manetti, Gemma Lepri, Silvia Bellando-Randone, Jelena Blagojevic, Lidia Ibba-Manneschi, Marco Matucci-Cerinic, Serena Guiducci

**Affiliations:** Department of Experimental and Clinical Medicine, Division of Rheumatology, Azienda Ospedaliero-Universitaria Careggi (AOUC), University of Florence, Viale Pieraccini 18, I-50139 Florence, Italy; Department of Experimental and Clinical Medicine, Section of Anatomy and Histology, University of Florence, Largo Brambilla 3, I-50134 Florence, Italy

## Abstract

**Introduction:**

The vascular and nervous systems have several anatomic and molecular mechanism similarities. Emerging evidence suggests that proteins involved in transmitting axonal guidance cues, including members of class III semaphorin (Sema3) family, play a critical role in blood vessel guidance during physiological and pathological vascular development. Sema3E is a natural antiangiogenic molecule that causes filopodial retraction in endothelial cells, inhibiting cell adhesion by disrupting integrin-mediated adhesive structures. The aim of the present study was to investigate whether in systemic sclerosis (SSc) Plexin-D1/Sema3E axis could be involved in the dysregulation of vascular tone control and angiogenesis.

**Methods:**

Sema3E levels were measured by quantitative colorimetric sandwich ELISA in serum samples from 48 SSc patients, 45 subjects with primary Raynaud's phenomenon (pRP) and 48 age-matched and sex-matched healthy controls. Immunofluorescence staining on skin sections from 14 SSc patients and 12 healthy subjects was performed to evaluate Sema3E and Plexin-D1 expression. Western blotting was used to assess Plexin-D1/Sema3E axis in human SSc and healthy dermal microvascular endothelial cells (SSc-MVECs and H-MVECs, respectively) at basal condition and after stimulation with recombinant human vascular endothelial growth factor (VEGF), SSc and healthy sera. Capillary morphogenesis on Matrigel was performed on H-MVECs treated with healthy, pRP or SSc sera in the presence of Sema3E and Plexin-D1 soluble peptides.

**Results:**

Serum Sema3E levels were significantly higher both in pRP subjects and SSc patients than in controls. In SSc, Sema3E levels were significantly increased in patients with early nailfold videocapillaroscopy (NVC) pattern compared to active/late patterns and pRP, and in patients without digital ulcers *versus* those with ulcers. In SSc skin, Sema3E expression was strongly increased in the microvascular endothelium. Cultured SSc-MVECs showed higher levels of phosphorylated Plexin-D1 and Sema3E expression than H-MVECs, and stimulation with SSc sera increased phosphorylated Plexin-D1 and Sema3E in H-MVECs. The addition of Sema3E-binding Plexin-D1 soluble peptide significantly attenuated the antiangiogenic effect of SSc sera on H-MVECs.

**Conclusions:**

Our findings suggest that Plexin-D1/Sema3E axis is triggered in SSc endothelium and may have a role in the dysregulation of angiogenesis and vascular tone control by inducing neuro-vascular mechanism alterations clinically evident in particular in the early disease phases.

## Introduction

Systemic sclerosis (SSc) is a multisystem autoimmune disease of unknown etiology characterized by vascular damage, activation of the immune system, and excessive deposition of collagen in the skin and internal organs including lungs, heart, gastrointestinal tract, and kidneys [1]. SSc is classified into limited cutaneous (lcSSc) and diffuse cutaneous (dcSSc) subsets and, in both, is associated with Raynaud's phenomenon (RP), nailfold capillaroscopic changes and antinuclear antibody positivity [2]. RP is characterized by recurrent, reversible episodes of vasospasm involving peripheral small vessels [3–5]. Early in the pathogenesis of RP, endothelial dysfunction increases platelet adhesion and favours dysfunctional control of vascular tone [6]. Endothelial cells are intimately involved in the regulation of vascular tone by synthesis and release of neurotransmitters, cytokines, growth factors, and prostaglandins, which mediate vasodilation and vasoconstriction [7]. Moreover, the vascular wall consists of endothelial, smooth muscle and fibroblast cells working in an integrated system of cellular paracrine/autocrine regulation interacting with the peripheral nervous system. The pathophysiology of RP is still not completely understood, but it has been suggested that dysregulation in neuro-endothelial control mechanisms plays a key role in RP pathogenesis [8].

**Fig. 1:**
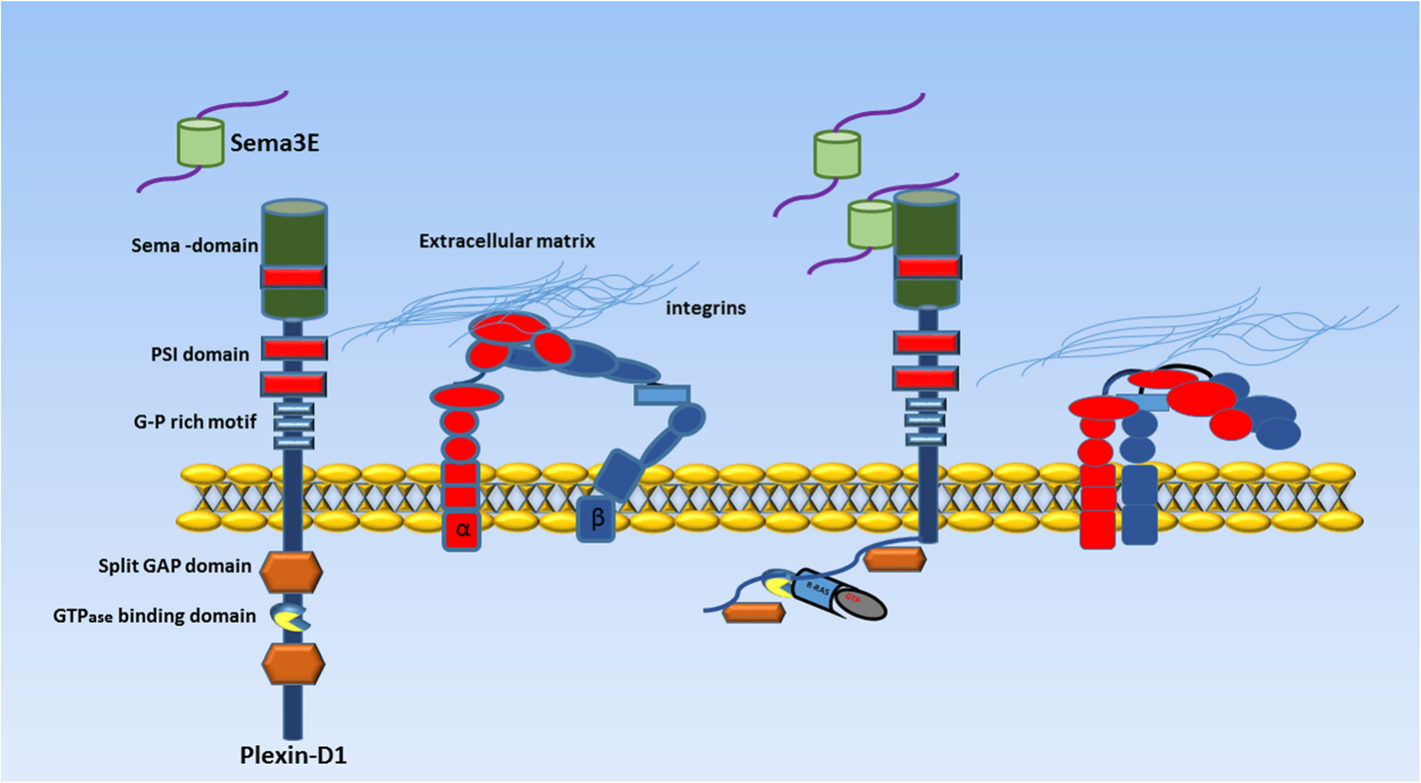
Schematic representation of Plexin-D1/Semaphorin 3E (Sema3E) axis in endothelial cells. Binding of Sema3E to the cell surface receptor Plexin-D1 activates an antiangiogenic signaling pathway leading to the disassembly of integrin-mediated focal adhesions, inhibition of endothelial cell adhesion to the extracellular matrix, and filopodia retraction in endothelial cells of growing blood vessels

Indeed, the vascular and nervous systems have several anatomical similarities that extend to molecular level, and the molecular mechanisms of nerve regulation are shared by the vascular system [9–12]. In some cases, vessels produce signals that attract axons to track alongside the pioneer vessels, conversely, nerves may also produce signals such as vascular endothelial growth factor (VEGF) to guide blood vessel growth. During embryogenesis, the developing nervous and vascular systems follow parallel routes and share several molecular pathways, including netrins, ephrins, and semaphorins, which act as both axon guidance molecules and regulators of developmental and postnatal angiogenesis [9, 13]. Among them, secreted class III semaphorins (Sema3s) control endothelial cell migration in vitro and in vivo [14, 15]. Semaphorins are grouped into eight classes based on their structural domains, and they are characterized by an amino-terminal Sema domain that is essential for signaling and can play a repulsive or attractive role depending on the cell types and biological context [16–18]. All Sema3 proteins (except Sema3E) signal through two major receptor families, namely Plexins and Neuropilins (NRPs), by forming a holoreceptor complex consisting of NRPs as ligand binding, and Plexins as signal transducing subunit. Conversely, Sema3E is unique in that it binds and signals directly through Plexin-D1 (PlxnD1), independently of NRPs. Plexins are single-pass transmembrane receptors subdivided into four groups, namely type A, B, C and D Plexins, with an extracellular Sema domain. Semaphorins target the actin cytoskeleton and focal adhesions, inducing cytoskeletal remodeling, cell motility, and cell migration [19, 20]. Furthermore, it has been shown that Sema3E acts on PlxnD1 in endothelial cells to start an antiangiogenic signaling pathway [21], through the disassembly of integrin-mediated focal adhesions and consequent inhibition of endothelial cell adhesion to the extracellular matrix, ultimately leading to filopodia retraction in endothelial cells of growing blood vessels (Fig. 1) [22]. Moreover, PlxnD1 was shown to be highly expressed in angiogenic endothelial cells, and it was found to regulate the morphogenic patterning of developing vascular networks [13].

On these premises, the aims of this study were to investigate 1) the possible contribution of the antiangiogenic PlxnD1/Sema3E pathway in angiogenesis disturbances and development of capillary abnormalities and digital ulcers (DUs) during SSc [23–26], and 2) if this axis might participate in the dysregulation of vascular tone control in both SSc and primary RP (pRP).

## Methods

### Study participants

Serum samples were obtained from 48 patients with SSc (43 female and 5 male patients; median age 64 years, range 37 to 78 years), defined as limited cutaneous SSc (lcSSc; n = 36) or diffuse cutaneous SSc (dcSSc; n = 12) [27], 45 subjects with pRP and 48 age-matched and sex-matched healthy controls. All SSc patients reported the occurrence of RP. Nailfold videocapillaroscopy (NVC) was performed on 10 fingers and all SSc patients were classified as having early, active and late NVC patterns [28]. At the time of blood collection, the presence of DUs was recorded. DUs related to trauma or calcinosis were not included in the analyses. The patients enrolled were not on corticosteroids, immunosuppressants or other disease-modifying drugs or calcium channel blockers. Peripheral blood samples were collected without any additive, left to clot for 30 minutes before centrifugation at 1,500 *g* for 15 minutes, and serum was collected and stored in aliquots at −80 °C until used. Paraffin-embedded sections of lesional forearm skin biopsies were obtained from 14 patients with SSc (12 women and 2 men, n = 9 with lcSSc and n = 5 with dcSSc; median age 46.8 years, range 27 to 69 years, and median disease duration 6 years, range 1 to 16 years) and 12 age-matched and sex-matched healthy donors. The study was approved by the Ethical Committee of the Azienda Ospedaliero-Universitaria Careggi (AOUC), Florence, Italy, and all subjects provided written informed consent.

### Isolation of dermal microvascular endothelial cells (MVECs) and cell culture

Human dermal MVECs were isolated from skin biopsies of five SSc patients (SSc-MVECs) and five healthy subjects (H-MVECs). Briefly, the samples were mechanically cleaned to remove the adipose and epidermis layers, in order to obtain a pure specimen of vascularized dermis, and were treated as previously described [29]. The samples were placed at 37 °C in a humidified atmosphere with 5 % CO_2_. After one day of culture in endothelial cell basal medium (EBM-2, catalog number LOCC3156; Euroclone, Milan, Italy) supplemented with 20 % fetal bovine serum (FBS), 5 ng/ml H-epidermal growth factor (hEGF; Clonetics Corporation, San Diego, California, USA), 1 μg/ml hydrocortisone acetate, 100 U/ml penicillin, 100 μg/ml streptomycin, and 25 μg/ml amphotericin B without addition of further angiogenic growth factors, small colonies of polygonal elements were detected. Non-adherent cells were removed and fresh endothelial cell complete medium was added. To maintain optimal culture conditions, media were changed every third day, and after 2 weeks of primary culture a monolayer of cells was obtained. MVECs from primary cultures were further identified using immunomagnetic beads recognizing CD31. Isolated cells were purified as MVECs by labeling with anti-factor VIII-related antigen and anti-CD105, followed by reprobing with anti-CD31 antibodies. Dermal MVECs were maintained in endothelial cell complete medium and were used between the third and seventh passages in culture.

### ELISA on serum samples

Serum levels of Sema3E protein were measured by a colorimetric sandwich enzyme-linked immunosorbent assay (ELISA kit, catalogue number ABIN1117868; Antibodies Online, Aachen, Germany), according to the manufacturer’s instructions. Briefly, standards and samples (100 μl/well) were added to the appropriate 96-well microtiter plate pre-coated with a biotin-conjugated polyclonal antibody specific for the short basic Sema3E domain, and were incubated for 2 hours at 37 °C. Subsequently the samples and standard were removed and 100 μl of Detection Reagent A working solution were added to each well for 1 hour at 37 °C. The microplates were washed three times with wash solution, followed by the addition of 100 μl of Detection Reagent B working solution (Avidin-conjugated Horseradish Peroxidase (HRP)) to each well, and incubation for 30 minutes at 37 °C. The microplates were washed five times and the reaction was developed in the dark with 90 μl of substrate solution (tetramethylbenzidine) and then stopped by applying 50 μl of sulfuric acid (1 M H_2_SO_4_). The absorbance of each well was read using a microplate reader at 450 nm. Serum levels of Sema3E were read from a standard curve prepared using a lyophilized protein standard reconstituted with standard diluent included in the kit. The detection range of the assay was 0.156−20 ng/ml. Each sample was measured in duplicate. Serum Sema3E concentration was determined by comparing the optical density (OD) of each sample to the standard curve.

### Immunohistochemistry and fluorescence microscopy

Paraffin-embedded skin sections (5 μm thick) were deparaffinized and boiled for 10 minutes in sodium citrate buffer (10 mM, pH 6.0) in order to expose antigens. After three washes in phosphate-buffered saline (PBS), the sections were incubated in 2 mg/ml glycine for 10 minutes to quench autofluorescence due to free aldehydes, and then blocked for 1 hour at room temperature with 1 % bovine serum albumin (BSA) in PBS. The samples were then incubated overnight at 4 °C with goat anti human Sema3E antibody (catalog number ab112886; Abcam, Cambridge, UK) diluted 1:100 in PBS with 1 % BSA. After extensive washing in PBS, the sections were incubated with Alexa Fluor-488-conjugated donkey anti-goat IgG for 45 minutes at room temperature in the dark (1:200 dilution; Invitrogen, San Diego, CA, USA). For double immunofluorescence staining, we used a rabbit polyclonal antibody against CD31/platelet-endothelial cell adhesion molecule-1 (PECAM-1) diluted 1:50 (catalog number ab28364; Abcam), followed by Alexa Fluor-568-conjugated donkey anti-rabbit IgG (1:200 dilution; Invitrogen). To evaluate PlxnD1 expression, skin sections were incubated overnight at 4 °C with rabbit polyclonal antibody against human PlxnD1 (catalog number ab28762; Abcam) diluted 1:50 in PBS with 1 % BSA. After extensive washing in PBS, the section were processed as described above, and subsequently incubated with Alexa Fluor-488-conjugated goat anti-rabbit IgG (1:200 dilution; Invitrogen) for 45 minutes at room temperature in the dark. For double immunofluorescence staining with anti-PlxnD1 antibody, we used a mouse monoclonal anti-CD31 antibody (1:20 dilution; catalog number M0823; Dako, Glostrup, Denmark) followed by Rhodamine Red-X-conjugated goat anti-mouse IgG (1:200 dilution; Invitrogen). Irrelevant isotype-matched and concentration-matched goat, mouse, and rabbit IgG (Sigma-Aldrich, St Louis, MO, USA) were used as negative controls. Nuclei were counterstained with 4′,6-diamidino-2-phenylindole (DAPI) (Chemicon International, Temecula, CA, USA). The immunolabeled sections were then observed under a Leica DM4000 B microscope equipped with fully automated fluorescence axes (Leica Microsystems, Mannheim, Germany). Fluorescence images were captured using a Leica DFC310 FX 1.4-megapixel digital colour camera equipped with the Leica software application suite LAS V3.8 (Leica Microsystems). Densitometric analysis of the intensity of immunofluorescent staining was performed on digitized images using the free-share ImageJ software (NIH, Bethesda, MD, USA; online at [30]).

### Western blotting and cell signaling

Total proteins were extracted from dermal H-MVECs and SSc-MVECs according to standard protocols. In some experimental conditions, before protein extraction H-MVECs were cultured for 24 hours in EBM-2 medium containing 10 % of serum from healthy subjects (n = 5) and SSc patients (n = 5) or recombinant human VEGF-A165 (10 ng/ml; R&D Systems, Minneapolis, MN, USA). As determined by ELISA, healthy and SSc serum samples containing Sema3E levels similar to the respective median group value were used in these experiments. Twenty-five micrograms of total proteins were electrophoresed on NuPAGE 4 to 12 % Bis-Tris Gel (Invitrogen) and were blotted on polyvinylidene difluoride membranes (Invitrogen). The membranes were blocked for 40 minutes at room temperature with the blocking solution included in the Western Breeze Chromogenic Western Blot Immunodetection Kit (Invitrogen) on a rotary shaker and incubated for 1 hour at room temperature with rabbit polyclonal antihuman PlxnD1 (Sema domain 250 kDa, catalog number PP4401; 1:1000 dilution; ECM Biosciences, Versailles, Woodford County, KY, USA), rabbit polyclonal antihuman PlxnD1 (Cytoplasmic domain 250 kDa, catalog number PP4421; 1:500 dilution; ECM Biosciences), rabbit polyclonal antihuman PlxnD1 (a.a. 1635–1647 C-ter region 250 kDa, catalog number PP4441; 1:500 dilution; ECM Biosciences), rabbit polyclonal antihuman Sema3E (N-terminal region 87 kDa, catalog number SP4461; 1:1000 dilution; ECM Biosciences) and rabbit polyclonal anti-α-tubulin (catalog number ab18251; 1:1000 dilution; Abcam) antibodies, assuming α-tubulin as control invariant protein. Immunodetection was performed as described in the Western Breeze Chromogenic Immunodetection protocol (Invitrogen). ImageJ software (NIH) was used for densitometric analysis of the bands and all values were normalized to α-tubulin.

### In vitro capillary morphogenesis assay

In vitro capillary morphogenesis assay was performed in 96-well plates covered with Matrigel (BD Biosciences, Milan, Italy). Matrigel (50 μl; 10–12 mg/ml) was pipetted into culture wells and polymerized for 30 minutes to 1 hour at 37 °C, as described elsewhere [31]. H-MVECs and SSc-MVECs (30 × 10^3^ cells/well) were incubated in EBM-2 medium containing 10 % FBS. In some experimental conditions, H-MVECs were incubated in EBM-2 medium with 10 % serum from healthy subjects (n = 5), SSc patients (n = 5) and pRP subjects (n = 5), alone or in combination with human Sema3E peptide (100 ng/ml; catalog number ab39320; Abcam), human PlxnD1 peptide (a.a. 1683–1694) (100 ng/ml; catalog number ab45701; Abcam) or both. As determined by ELISA, healthy, pRP and SSc serum samples containing Sema3E levels similar to the respective median group value were used in these experiments. Stimulation with recombinant human VEGF-A165 (10 ng/ml; R&D Systems) was used as positive control of angiogenesis. Plates were photographed at 6 and 24 hours. Results were quantified at 24 hours by measuring the percent field occupancy of capillary projections, as determined by image analysis. Six to nine photographic fields from three plates were scanned for each experimental point.

### Statistical analysis

Data are expressed as the mean ± standard deviation (SD) or median and range. The Student’s *t* test and nonparametric Mann–Whitney *U* test were used where appropriate for statistical evaluation of the differences between two independent groups. A *p* value less than 0.05 was considered statistically significant.

## Results

### Serum levels of Sema3E

**Fig. 2:**
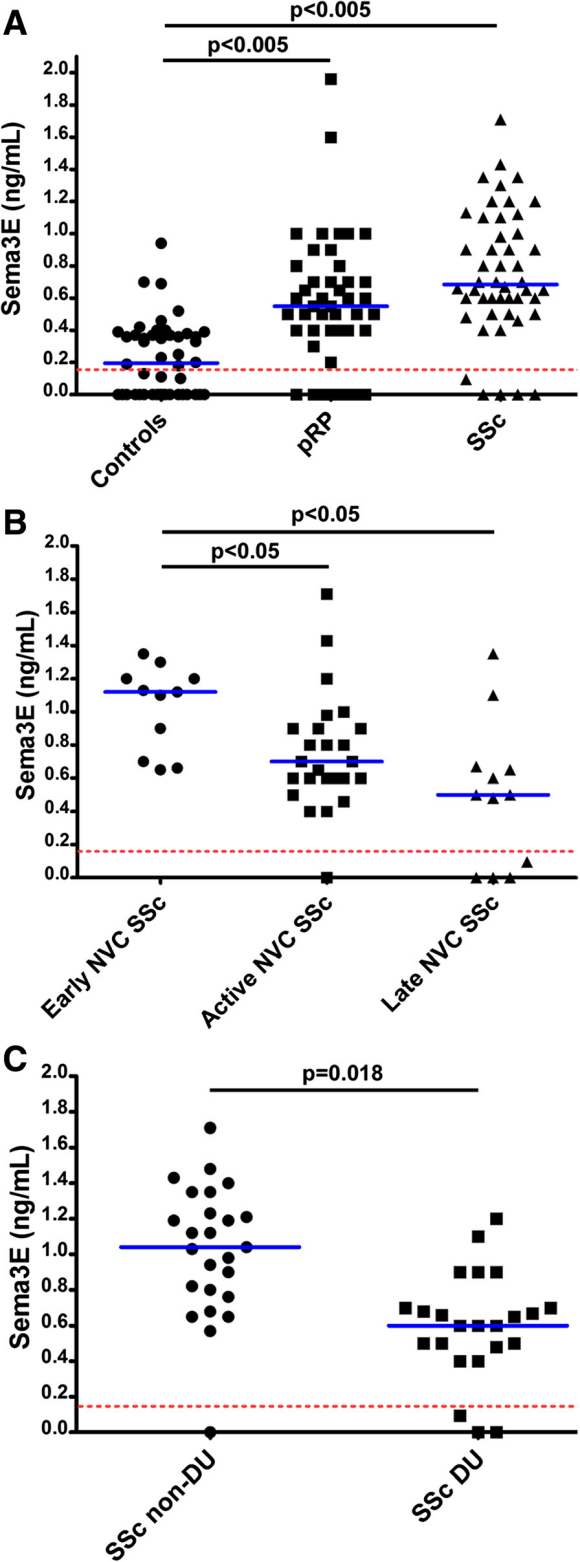
Serum levels of semaphorin 3E (*Sema3E*) determined by colorimetric sandwich enzyme-linked immunosorbent assay (ELISA). **a** Serum Sema3E levels in healthy controls, subjects with primary Raynaud's phenomenon (*pRP*) and patients with systemic sclerosis (*SSc*). **b** Serum Sema3E levels in SSc patients according to nailfold videocapillaroscopy (*NVC*) pattern (early, active and late). **c** Serum Sema3E levels in SSc patients according to the absence/presence of digital ulcers (*DUs*). Data are shown as dot plots. Each dot represents a subject. *Blue horizontal lines* indicate the median value in each group. In each panel, the *red dashed line* indicates the detection threshold of the ELISA. The nonparametric Mann–Whitney *U* test for independent samples was used to analyze the serum Sema3E differences between groups. Values of *p* <0.05 were considered significant; *p* values are indicated in each panel

Sema3E serum levels were significantly increased both in pRP subjects (median 0.54 ng/ml, range 0.00−1.96 ng/ml) and SSc patients (median 0.67 ng/ml, range 0.00−1.71 ng/ml) compared to controls (median 0.19 ng/ml, range 0.00−0.94 ng/ml) (both *p* <0.005) (Fig. 2a). No significant differences in circulating Sema3E levels were detected between pRP and SSc (Fig. 2a). Next, we correlated serum Sema3E levels with NVC patterns as measure of peripheral microvascular involvement. In SSc patients with early NVC pattern, Sema3E levels were significantly increased (median 1.17 ng/ml, range 0.65−1.35 ng/ml) compared with active (median 0.65 ng/ml, range 0.00−1.71 ng/ml) and late (median 0.55 ng/ml, range 0.00−1.35 ng/ml) NVC patterns (both *p* <0.05) (Fig. 2b). Moreover, serum Sema3E in SSc patients with early NVC pattern was significantly higher than in pRP subjects (median 0.54 ng/ml, range 0.00−1.96 ng/ml) (*p* <0.05). In addition, Sema3E levels were significantly increased in SSc patients without DUs (median 1.03 ng/ml, range 0.00−1.71 ng/ml) compared with patients with DUs (median 0.57 ng/ml, range 0.00−1.20 ng/ml) (*p* = 0.018) (Fig. 2c).

### Expression of Sema3E and PlxnD1 in skin biopsies

**Fig. 3:**
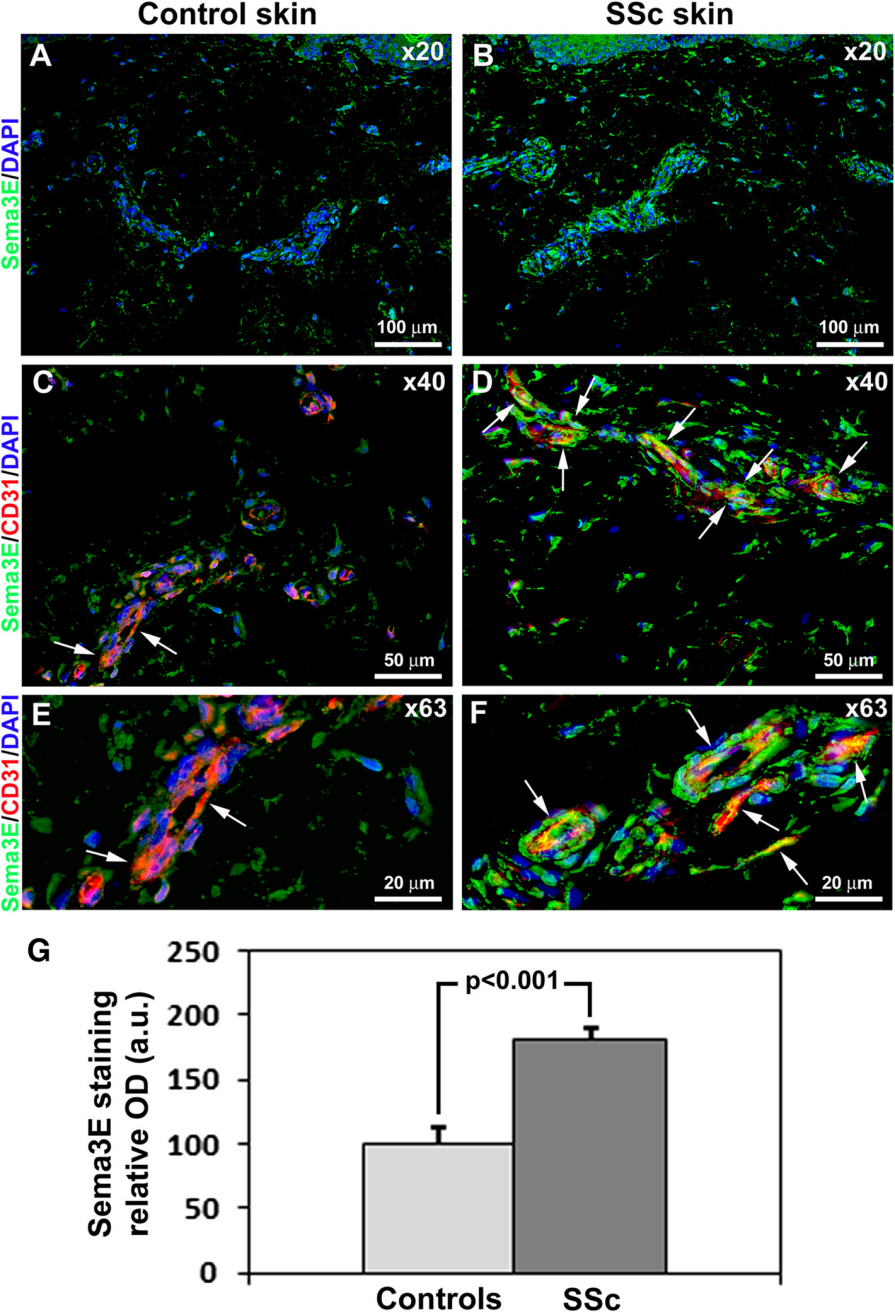
Expression of semaphorin 3E (*Sema3E*) in skin of healthy controls and patients with systemic sclerosis (*SSc*). **a**, **b** Representative microphotographs of skin sections from healthy controls (**a**) (n = 12) and patients with SSc (**b**) (n = 14) immunostained for Sema3E (*green*) and counterstained with 4′,6-diamidino-2-phenylindole (*DAPI*; *blue*) for nuclei. **c**-**f** Representative microphotographs of skin sections from healthy controls (**c**, **e**) (n = 12) and patients with SSc (**d**, **f**) (n = 14) double immunostained for Sema3E (*green*) and CD31/platelet-endothelial cell adhesion molecule-1 (PECAM-1) (*red*) and counterstained with DAPI (*blue*). *Arrows* indicate Sema3E expression in dermal microvascular endothelial cells. Original magnification: × 20 (**a**, **b**), × 40 (**c**, **d**), × 63 (**e**, **f**). Scale bars are indicated in each panel. **g** Densitometric analysis of Sema3E immunofluorescent staining in skin biopsies expressed as optical density (*OD*) in arbitrary units (*a.u.*). Control skin OD value was set to 100 %; the other results are normalized to this value. Data are mean ± SD. Student’s *t* test was used for statistical analysis

**Fig. 4:**
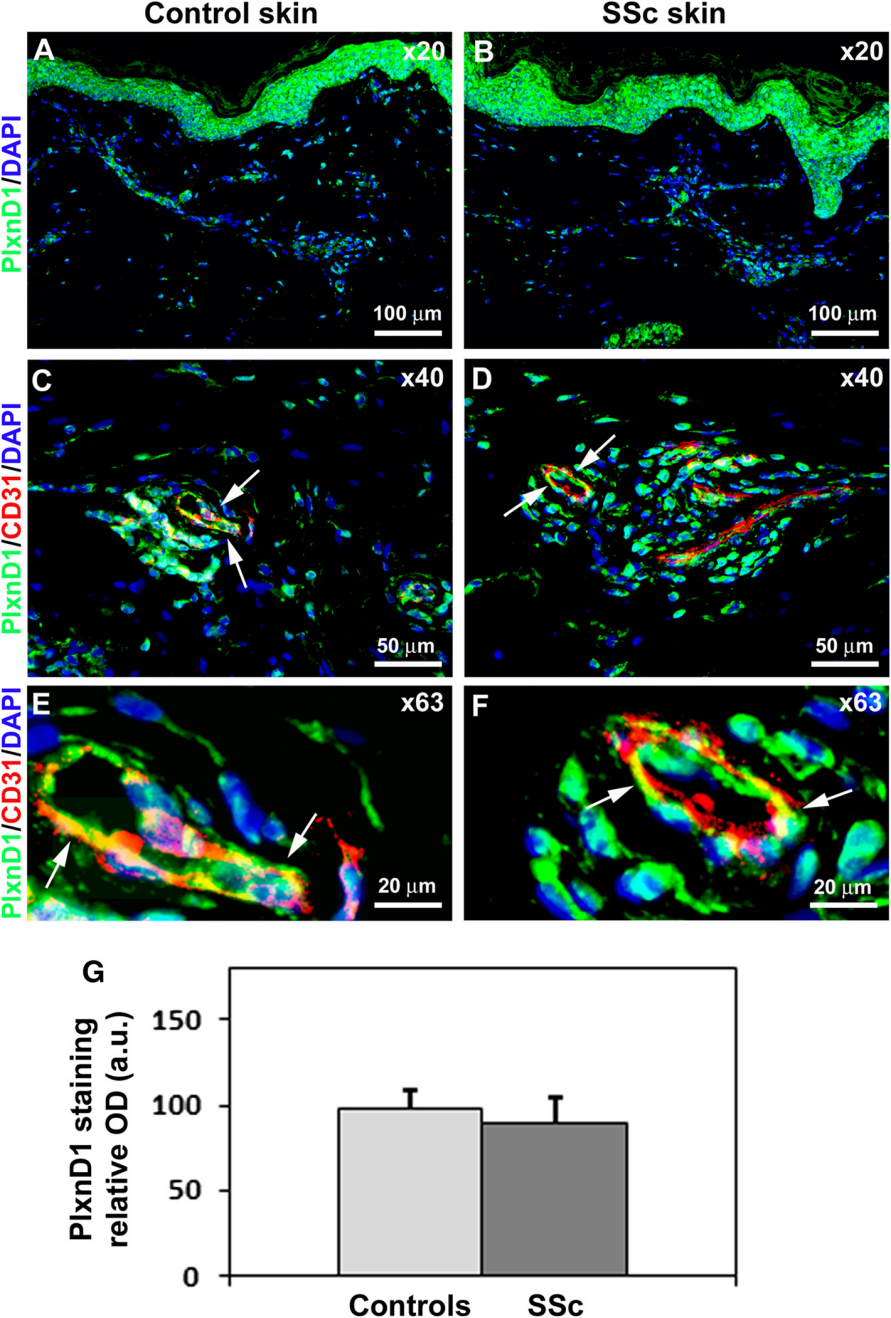
Expression of Plexin-D1 (*PlxnD1*) in skin of healthy controls and patients with systemic sclerosis (*SSc*). **a**, **b** Representative microphotographs of skin sections from healthy controls (**a**) (n = 12) and SSc patients (**b**) (n = 14) immunostained for PlxnD1 (*green*) and counterstained with 4′,6-diamidino-2-phenylindole (*DAPI*; *blue*) for nuclei. **c**-**f** Representative microphotographs of skin sections from healthy controls (**c**, **e**) (n = 12) and SSc patients (**d**, **f**) (n = 14) double immunostained for PlxnD1 (*green*) and CD31/platelet-endothelial cell adhesion molecule-1 (PECAM-1) (*red*) and counterstained with DAPI (*blue*). *Arrows* indicate PlxnD1 expression in dermal microvascular endothelial cells. Original magnification: × 20 (**a**, **b**), × 40 (**c**, **d**), × 63 (**e**, **f**). Scale bars are indicated in each panel. **g** Densitometric analysis of PlxnD1 immunofluorescent staining in skin biopsies expressed as optical density (*OD*) in arbitrary units (*a.u.*). Control skin OD value was set to 100 %; the other results are normalized to this value. Data are mean ± SD. Student’s *t* test was used for statistical analysis

The expression of Sema3E and its receptor PlxnD1 in skin biopsies from SSc patients and healthy controls was evaluated by immunofluorescence (Figs. 3 and 4). Sema3E expression was strongly increased in several cellular components of SSc dermis, such as fibroblasts and perivascular cells when compared with healthy controls (Fig. 3a, b). Moreover, double immunofluorescence staining for Sema3E and the pan-endothelial cell marker CD31/PECAM-1 revealed that Sema3E expression was significantly upregulated in endothelial cells of SSc dermis compared with healthy control skin (Fig. 3c -f). The densitometric analysis of the intensity of immunofluorescent staining demonstrated that Sema3E expression was significantly increased in the dermis of SSc patients compared with controls (*p* <0.001) (Fig. 3g). Conversely, no significant difference in the expression of PlxnD1 was observed between healthy skin and skin from patients with SSc in different dermal cell types, including endothelial cells (Fig. 4a-g).

### PlxnD1/Sema3E cell signaling in dermal MVECs

**Fig. 5:**
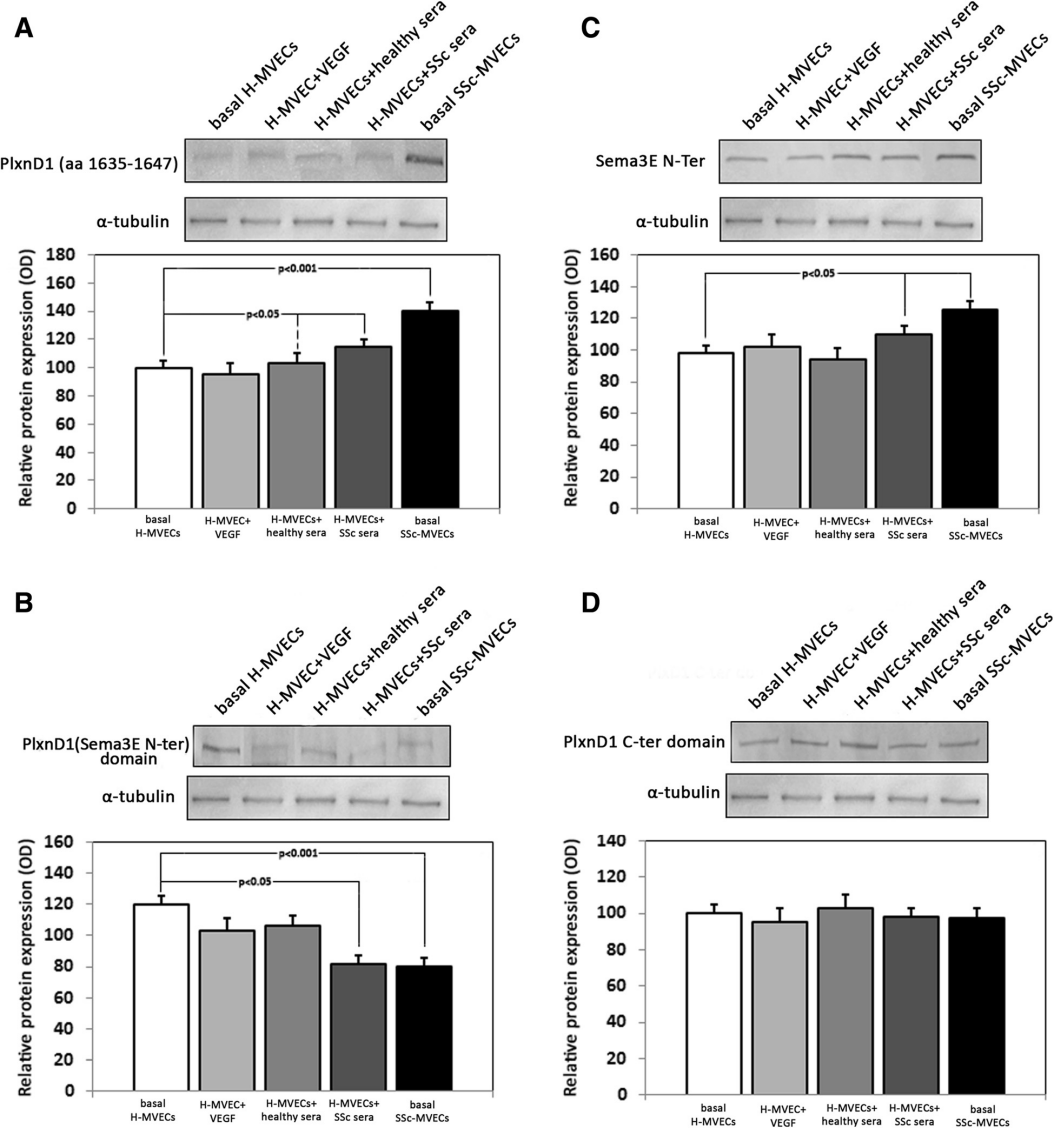
Differential activation of Plexin-D1 (*PlxnD1*)**/**semaphorin 3E (*Sema3E*) cell signaling pathway in dermal microvascular endothelial cells (*MVECs*) from healthy controls (*H*) and patients with systemic sclerosis (*SSc*). Protein lysates from H-MVECs at basal condition and after stimulation with recombinant human vascular endothelial growth factor (*VEGF*)-A165, sera from healthy controls and patients with SSc (both n = 5), and from SSc-MVECs at basal condition were assayed with antibodies recognizing (**a**) the activated form of PlxnD1 (phosphorylated in C-ter domain, a.a. 1635–1647), (**b**) the N-terminal Sema domain region of PlxnD1, (**c**) Sema3E N-terminal region, and (**d**) PlxnD1 C-terminal domain. Representative immunoblots are shown. The densitometric analysis of the bands normalized to α-tubulin is reported in the histograms. Data are mean ± SD of optical density (*OD*) in arbitrary units. Student’s *t* test was used for statistical analysis; *p* values are indicated in each panel. Results are representative of three independent experiments performed with each one of the five H-MVEC and five SSc-MVEC lines

To assess the role of PlxnD1/Sema3E pathway in the endothelium, we evaluated cell signaling on H-MVECs cultured in standard condition and after challenge with recombinant human VEGF-A165 and sera from healthy subjects or patients with SSc, and in SSc-MVECs cultured in standard conditions. Western blotting analyses revealed that SSc-MVECs expressed significant higher levels of the activated form of PlxnD1 (phosphorylated in C-ter domain, a.a. 1635–1647) than H-MVECs (*p* <0.001) (Fig. 5a). In addition, the phosphorylated form of PlxnD1 significantly increased in H-MVECs challenged with sera from patients with SSc compared with both basal H-MVECs and H-MVECs cultured with sera from healthy controls (both *p* <0.05) (Fig. 5a). To validate these results, we also specifically evaluated the expression levels of the N-terminal Sema domain region of PlxnD1, which can be detected only when this domain is not bound by Sema3E. As shown in Fig. 5b, the detection levels of this region were significantly lower in SSc-MVECs than in H-MVECs, and in H-MVECs challenged with sera from patients with SSc compared with H-MVECs treated with sera from healthy controls (both *p* <0.05). Moreover, consistent with the immunohistochemical results, Sema3E levels were significantly increased in SSc-MVECs in respect to H-MVECs, and in H-MVECs challenged with sera from patients with SSc compared with H-MVECs treated with sera from healthy controls (both *p* <0.05) (Fig. 5c). No significant differences in total PlxnD1 expression were found between the different experimental conditions (Fig. 5d). Finally, the stimulation with recombinant human VEGF-A165 did not significantly affect the PlxnD1/Sema3E pathway in H-MVECs (Fig. 5a-d).

### Capillary morphogenesis on Matrigel

**Fig. 6:**
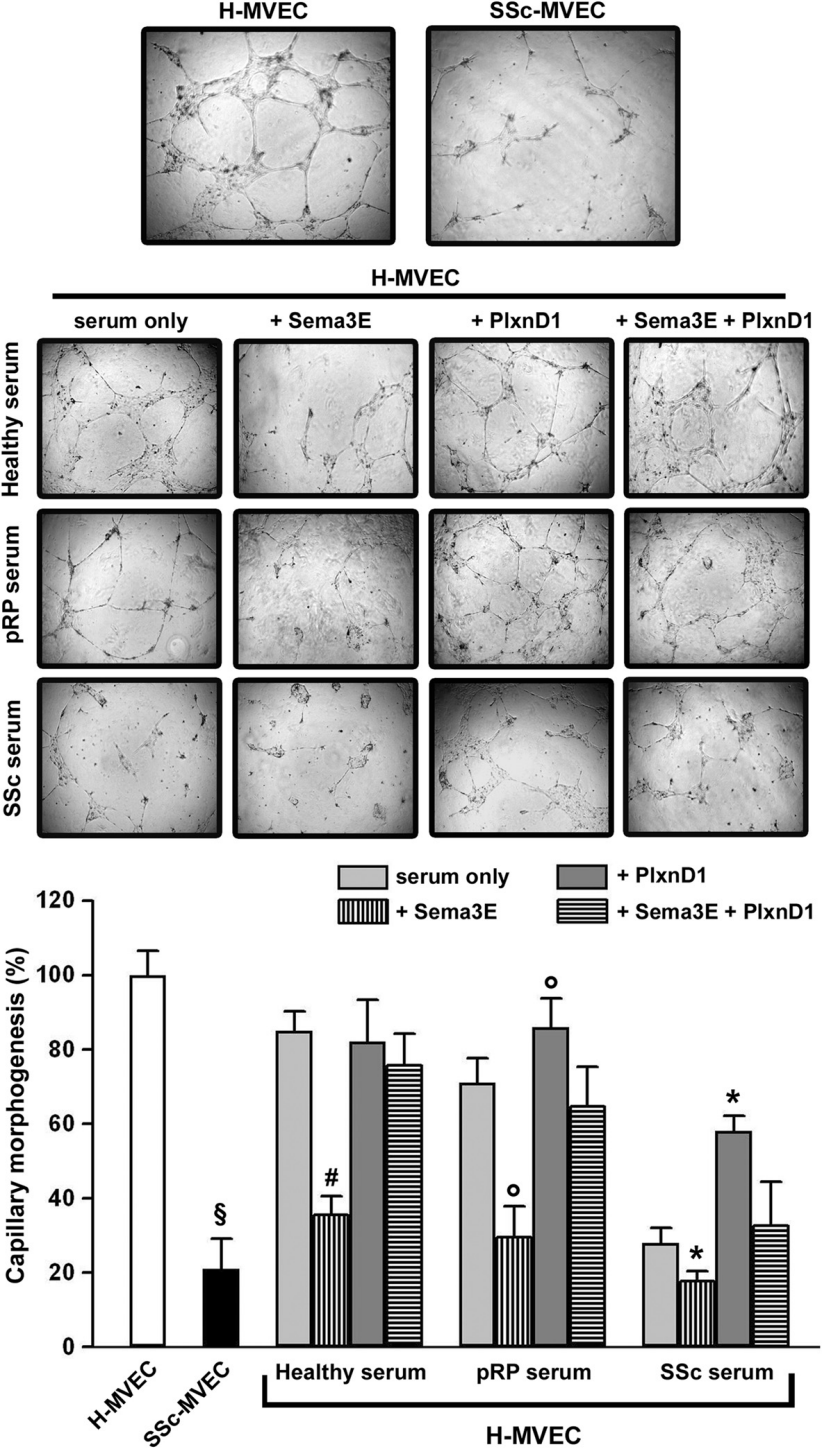
In vitro angiogenesis assay results. *Upper panel*: representative images of capillary morphogenesis on Matrigel after 24 hours. *Lower panel*: capillary morphogenesis of healthy and systemic sclerosis (*SSc*) dermal microvascular endothelial cells (*H-MVECs* and *SSc-MVECs*, respectively) quantified as percent field occupancy of capillary projections. Capillary morphogenesis of H-MVECs was evaluated at basal condition and after stimulation with sera from healthy subjects (n = 5), primary Raynaud's phenomenon (*pRP*) subjects (n = 5), and SSc patients (n = 5) alone or in combination with human semaphorin 3E (*Sema3E*) peptide, human Sema3E-binding Plexin-D1 (*PlxnD1*) peptide (a.a. 1683–1694), or both. Capillary morphogenesis of H-MVECs at basal condition was set to 100 %; the other results are normalized to this value. Data are the mean ± SD of three independent experiments performed in triplicate with each one of the five H-MVEC and five SSc-MVEC lines. Six to nine photographic fields from three plates were scanned for each experimental point. Student’s *t* test was used for statistical analysis. ^§^ *p* <0.05 *vs* basal H-MVECs; ^#^ *p* <0.05 *vs* healthy serum only; °*p* <0.05 *vs* serum from patients with pRP only; *p <0.05 *vs* serum from patients with SSc only

To investigate the functional influence of the PlxnD1/Sema3E pathway on dermal MVEC in vitro angiogenesis, we performed capillary morphogenesis on Matrigel matrix. In this assay, MVECs usually produce elongated processes that eventually form anastomosing cords of cells mimicking a tubular capillary plexus. Consistent with previous findings [31], in standard conditions angiogenesis was strongly impaired in SSc-MVECs compared with H-MVECs (Fig. 6). H-MVECs stimulated with healthy sera produced an abundant network of branching cords, while the addition of Sema3E soluble peptide to the sera significantly inhibited angiogenesis (*p* <0.05 *vs* healthy serum only) (Fig. 6). Furthermore, the angiogenic capacity of H-MVECs co-incubated with healthy sera and Sema3E-binding PlxnD1 peptide or Sema3E/PlxnD1 combined peptides was not different compared to that of cells treated with healthy sera alone (Fig. 6). Similar results were obtained when H-MVECs were challenged with sera from patients with pRP, except that co-incubation with pRP serum and Sema3E-binding PlxnD1 peptide significantly increased angiogenesis compared to treatment with serum from pRP alone (*p* <0.05) (Fig. 6). Moreover, capillary morphogenesis was reduced in H-MVECs challenged with sera from patients with pRP compared with cells treated with healthy sera, but this difference was not statistically significant. Conversely, treatment with sera from patients with SSc strongly reduced angiogenesis compared with healthy sera (Fig. 6). The addition of Sema3E peptide further impaired the angiogenic capacity of H-MVECs challenged with sera from patients with SSc (*p* <0.05 vs serum from patients with SSc only) (Fig. 6). On the contrary, when H-MVECs were co-incubated with sera from patients with SSc and Sema3E-binding PlxnD1 soluble peptide, their angiogenic capacity was significantly increased compared with cells treated with sera from patients with SSc alone (*p* <0.05) (Fig. 6).

## Discussion

Our data show for the first time that 1) serum levels of Sema3E are significantly increased both in pRP subjects and SSc patients with secondary RP, and that 2) the PlxnD1/Sema3E signaling pathway is triggered in SSc-MVECs and determines an antiangiogenic response.

Blood vessels provide oxygen and nutrients to every part of the body, and they are essential for tissue homeostasis and repair. Angiogenesis is a physiological process where new vessels are formed by sprouting of endothelial cells from pre-existent vessels to create and increase a complex network [32–35]. Angiogenesis is controlled by a tight and complex balance between proangiogenic and antiangiogenic signals, and among them VEGF coordinates the angiogenesis process. Moreover, the endothelium produces a large number of vasodilating factors, including nitric oxide and prostacyclin [36], and vasoconstricting substances, such as endothelin-1, with effects on vascular remodeling [37]. In this context, increasing evidence indicates that both pRP and secondary RP in SSc are characterized by severe disturbances in multiple mechanisms that regulate vascular tone control [2, 24]. Moreover, an impaired angiogenic response following tissue ischemia and hypoxia is considered a hallmark feature of SSc [24].

Of note, it is well-established that the autonomic nervous system contributes to the pathogenesis of both pRP and secondary RP in SSc, via central and peripheral mechanisms [38]. Furthermore, the vascular and nervous systems have several morphological and molecular similarities [9, 19, 35]. In fact, emerging evidence suggests that proteins involved in transmitting axonal guidance cues, such as the multiple members of secreted class III semaphorin family that regulate developmental axonal growth, also play a critical role in blood vessel guidance during physiological and pathological vascular development [9, 39, 40]. In particular, soluble Sema3E is a potent inhibitor of angiogenesis that binds to its specific receptor PlxnD1 with consequent activation of a complex antiangiogenic intracellular signaling cascade [23].

The present study was undertaken to investigate whether the PlxnD1/Sema3E axis could be involved in the neurovascular component of SSc pathophysiology. For this purpose, we first analyzed circulating levels of Sema3E both in pRP subjects and SSc patients, as well as their possible correlation with measures of vascular involvement in SSc. Interestingly, we found significantly increased serum Sema3E levels both in pRP and SSc compared with healthy individuals, suggesting that this molecule might participate in the vascular tone disturbances characteristic of both clinical entities. Moreover, it is interesting to note that in SSc increased circulating levels of Sema3E specifically correlated with the early NVC pattern and the absence of DUs, as patients with more severe capillary damage and DUs had Sema3E levels comparable to those of controls. Thus, it is tempting to speculate that Sema3E might even serve in the future as a biomarker of early vascular involvement during SSc. Indeed, currently there are no reliable serological biomarkers for the early diagnosis of RP and the neuro-vascular involvement in SSc patients. However, further follow-up studies will be necessary to ascertain whether circulating Sema3E could be a useful marker to monitor the evolution of SSc-related peripheral vascular disease. Furthermore, we cannot exclude the possibility that lower Sema3E levels detected in SSc with more advanced NVC patterns might be either a cause or a consequence of the disease, which is characterized by progressive loss of the peripheral microvessels and nervous fibers [41], which are the main source of Sema3E.

Consistent with serum findings, the expression of Sema3E in SSc-affected dermis was strongly increased, particularly in the microvascular endothelium. Conversely, no difference in the expression of Sema3E receptor PlxnD1 was found between skin from patients with SSc and healthy controls. To further address whether the PlxnD1/Sema3E pathway could be activated in the endothelium in SSc, we performed cell signaling studies on cultured dermal MVECs. Strikingly, our in vitro experiments demonstrated that although total PlxnD1 expression was not different in H-MVECs and SSc-MVECs, these latter showed a significantly increase in the expression of the activated form of PlxnD1 (phosphorylated in C-ter domain). Moreover, phosphorylated PlxnD1 significantly increased in H-MVECs after challenge with sera from patients with SSc. Thus, these data support the hypothesis that PlxnD1/Sema3E pathway is triggered in the microvascular endothelium in SSc. Finally, functional capillary morphogenesis experiments revealed that such activation of the PlxnD1/Sema3E pathway may contribute to defective angiogenesis in SSc. Indeed, we showed that Sema3E exerts antiangiogenic effects in vitro. Accordingly, serum from patients with SSc strongly inhibited angiogenesis of H-MVECs, while the addition of Sema3E-binding PlxnD1 soluble peptide to sequester elevated Sema3E present in sera from patients with SSc significantly improved H-MVEC angiogenesis.

## Conclusions

In conclusion, our findings suggest that the PlxnD1/Sema3E axis is triggered in the endothelium in SSc, and may have a role in the dysregulation of angiogenesis and vascular tone control by inducing neurovascular mechanism alterations, which are clinically evident in particular in the early disease phases.

## Abbreviations

a.u.: arbitrary units
BSA: bovine serum albumin
DAPI: 4′,6-diamidino-2-phenylindole
dcSSc: diffuse cutaneous systemic sclerosis
DU: digital ulcer
EBM: endothelial cell basal medium
ELISA: enzyme-linked immunosorbent assay
H-MVECs: human dermal microvascular endothelial cells from healthy subjects
IgG: immunoglobulin G
lcSSc: limited cutaneous systemic sclerosis
MVECs: microvascular endothelial cells
NRP: neuropilin
NVC: nailfold videocapillaroscopy
OD: optical density
PBS: phosphate-buffered saline
PECAM-1: platelet-endothelial cell adhesion molecule-1
PlxnD1: Plexin-D1
pRP: primary Raynaud’s phenomenon
RP: Raynaud’s phenomenon
SD: standard deviation
Sema3E: semaphorin 3E
SSc: systemic sclerosis
SSc-MVECs: human dermal microvascular endothelial cells from patients with systemic sclerosis
VEGF: vascular endothelial growth factor
